# A sulfated exopolysaccharide derived from *Chlorella* sp. exhibiting in vitro anti*-α-*d*-*Glucosidase activity

**DOI:** 10.1007/s00203-024-03940-6

**Published:** 2024-04-16

**Authors:** Karima Guehaz, Zakaria Boual, Alia Telli, Hicham Meskher, Hakim Belkhalfa, Guillaume Pierre, Philippe Michaud, Alessandra Adessi

**Affiliations:** 1 Laboratory for the Protection of Ecosystems in Arid and Semi-Arid Zones, FNSV, Kasdi Merbah University, 30000 Ouargla, Algeria; 2 Division of Process Engineering, College of Science and Technology, Chadli Bendjedid University, 36000 El Tarf, Algeria; 3 Scientific and Technical Research Center in Physicochemical Analysis, 30000 Ouargla, Algeria; 4 Institut Pascal, Université Clermont Auvergne, CNRS, Clermont Auvergne INP, 63000 Clermont-Ferrand, France; 5 Department of Agriculture Food Environment and Forestry (DAGRI), University of Florence, 50144 Florence, Italy

**Keywords:** *Chlorella* sp., Exopolysaccharide, Salt lake, *α-*d*-*Glucosidase inhibition

## Abstract

There is a great scientific curiosity to discover all environments sheltering microalgae, especially those with exceptional characteristics from coldest to hottest ones, the purpose remains to explore the potential of the native microalgae flora and the research for new bioactive compounds. This study aimed to isolate a polysaccharide-producing microalga from an extreme ecosystem and to evaluate its capacity to inhibit the *α-*d*-*glucosidase enzyme. *Chlorella* strain is isolated from hypersaline Lake in the Algerian desert. The exopolysaccharide extraction was performed by the concentration of free-cell supernatant in a rotary evaporator. The infrared analysis showed a characteristic footprint of carbohydrates with particular functional groups, such as sulfate. Gas chromatography–mass spectrometry has revealed a hetero-exopolysaccharide composed of galactose 35.75%, glucose 21.13%, xylose 16.81%, fructose 6.96%, arabinose 5.10%, and glucuronic acid 2.68%. The evaluation of the anti-hyperglycemic activity demonstrated a significant α-d-glucosidase inhibition of 80.94 ± 0.01% at 10 mg mL^−1^ with IC_50_ equal to 4.31 ± 0.20 mg mL^−1^. This study opens a vast prospect to use exopolysaccharides as natural nutraceutical or food additive.

**Graphical Abstract:**

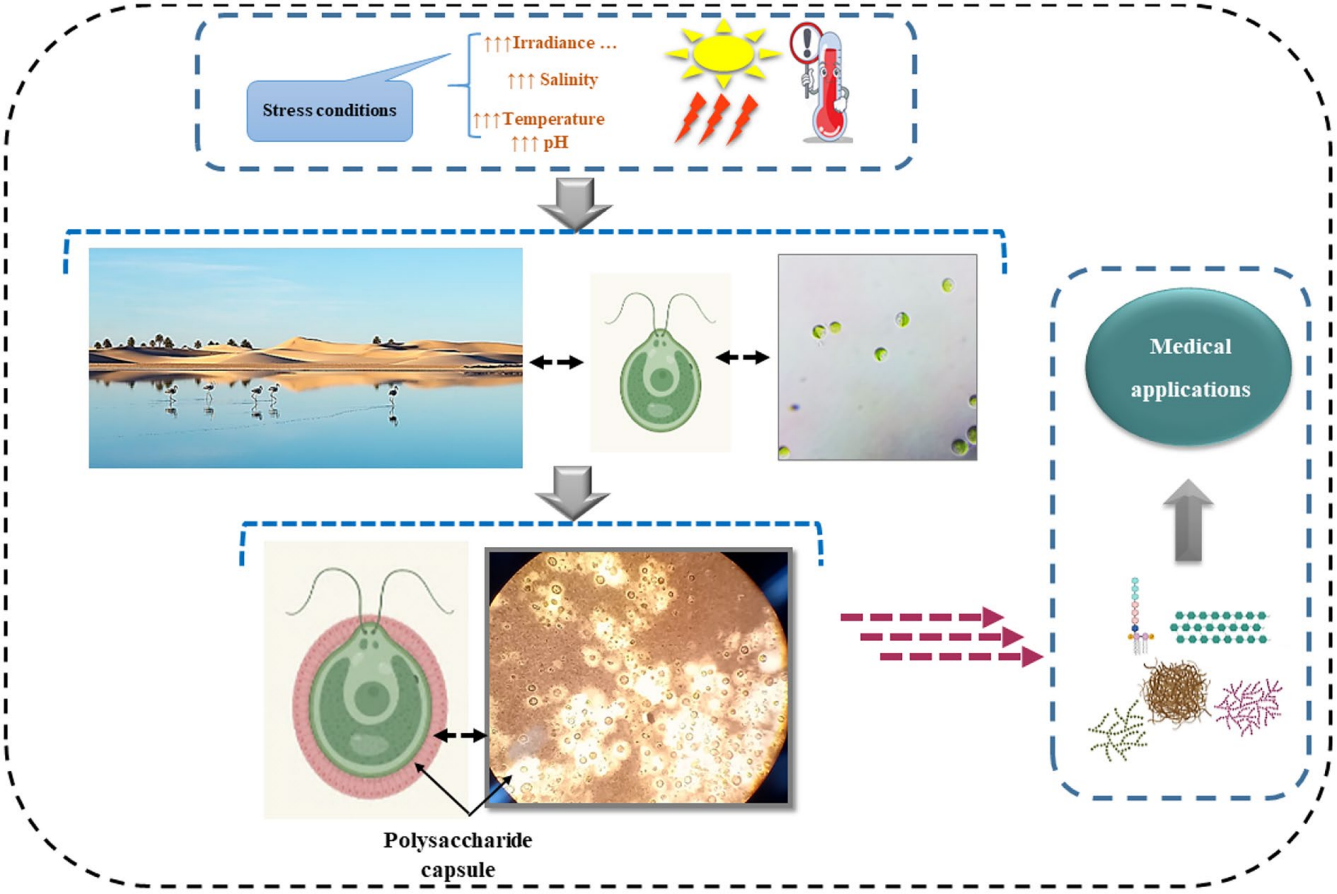

## Introduction

The scientific community continues exploring microalgae molecules from new environments, aiming to find bioactive compounds with interesting properties to use in biotechnology. Microalgae are photosynthetic organisms known for their extraordinary diversity, and natural origin, making them a good choice for a variety of applications (Colusse et al. 2022).

Green microalgae have been receiving increased attention since they present the most diverse group of microalgae. Among Chlorophycean genera; *Chlorella* is a small, single-celled, green, eukaryotic microalga, with a size around 2–15 μm in diameter. These unicellular green microalgae are present in all water habitats (fresh and marine water), exhibiting a cosmopolitan occurrence (Wong et al. 2015). Despite being a simple alga, *Chlorella* is a rich source of a variety of molecules implicated in a large spectrum of innovative applications such as bioremediation, biofuels, food, feed, biopolymers, and aquaculture (Abreu et al. 2023).

*Chlorella* is a potent microalga that possesses excellent potential, some species have been approved for human consumption by the European Food Safety Authority (No 258/97)^1^ (Niccolai et al. 2019). Its supplementation in mammals, including humans, has been reported to exhibit various pharmacological activities (Neumann et al. 2018; Ramos-Romero et al. 2021). Therefore, *Chlorella* species have been added to different commercial food products as dietary supplements. It has been incorporated as whole biomass to ameliorate the techno-functional and nutritional properties in pasta, yogurt, cheese, cookies, and water/oil emulsions (Caporgno and Mathys 2018).

*Chlorella *is among the few eukaryotic microalgae that synthesize substances with a broad spectrum of antibacterial/antifungal activity (Mashhadinejad et al. 2016; Dinev et al. 2021; Shaima et al. 2022), *Chlorella marina *produces lycopene (Mtaki et al. 2020), *Chlorella stigmatophora *produces polysaccharides (Sathasivam et al. 2019), *Chlorella pyrenoidosa *produces flavonoids (Yadavalli et al. 2022), *Chlorella protothecoides* produces sporopollenin (He et al. 2016), *Chlorella fusca* produces sporopollenin (Priyadarshani and Rath 2012). Furthermore, *Chlorella* produces a plethora of biomolecules with relevant properties for human health and food industry, such as polysaccharides (PSs). PSs are considered the primary active component of *Chlorella* owning diverse health-promoting potentials and food functional qualities, like those associated with antioxidation, anticoagulation, immunomodulation, anti-hyperlipidemia, antitumor, neuroprotection, and anti-asthmatic effect (Sheng et al. 2007; Chen et al. 2014, 2016; Barboríková et al. 2019; Wan et al. 2020; Mousavian et al. 2022; Tang et al. 2023).

Studies on the anti-diabetic activity of PSs derived from *Chlorella* are rare, most work done concerns the supply of *Chlorella* as whole biomass in rats and the evaluation of its activity (Jong-Yuh and Mei-Fen 2005; Senthilkumar and Ashokkumar 2012; Yuan et al. 2020; Ramos-Romero et al. 2021; Xiong et al. 2022). Qiu et al. (2022) have described the anti-hyperglycemic, antioxidant, and anti-inflammatory potential of PSs extracted from *Chlorella pyrenoidosa* in aging-related diabetes mice model. The suggested findings demonstrated the inhibition of high glucose levels through induction of insulin secretion, and the prevention of oxidation by improving the secretion of antioxidant enzymes (Qiu et al. 2022). The mechanisms by which *Chlorella* may protect humans from diabetes and related risk factors are mainly unclear, the common explanation given is that the protective effect against diabetes by inducing insulin secretion, but also by increasing the circulating glucose uptake in the liver and muscles (Bito et al. 2020; Ramos-Romero et al. 2021).

Most studies have used *Chlorella* as whole biomass in animal models for the assessment of the biological activities, thus, a synergetic effect between all compositional compounds occurs in the observed potential, and the main molecule responsible for the activity is not defined. Herein, there is a serious need to explore the bioactivity of each single metabolite. Therefore, this paper is a contribution to the evaluation of the antihyperglycemic activity through the assessment of α-d-glucosidase enzyme inhibition by exopolysaccharide (EPS) derived from a wild *Chlorella* sp. isolated from a hypersaline Lake (Chott Ain El-Beida), Ouargla-Algeria. The Lake is of international importance since it has been classified as an internationally protected wetland according to RAMSAR Convention 1971. The native microalgae flora in Algeria is still under-investigated, this is the first report on microalgae’s EPS derived from a local strain. To the best of our knowledge, no papers were reported on the evaluation of α-d-glucosidase inhibition by exopolysaccharide-producing *Chlorella* sp. from Saharan biotopes.

## Materials and methods

### Measurement of physicochemical parameters

A water sample was taken from Chott Aïn El-Beida lake Ouargla-Algeria (31°57′45.3″N 5°22′30.7″E) January 29th 2019, and the physicochemical parameters of water were measured using a multi-parameter analyzer; the parameters were: the hydrogen potential (pH 0.00–14.00), the temperature (°C), the salinity (g L^−1^), the conductivity (m S^−1^ cm^−1^), the dissolved oxygen (ppm) and the total dissolved solids (ppt) (HANNA HI 9828).

### Isolation and microscopic observation

The isolation of microalgae was accomplished by transferring the water sample into a solid Bold’s Basal media (BBM), at 1.5% of agar; macronutrients (g L^−1^ dH_2_O) NaNO_3_ 25, CaCl_2_.2H_2_O 2.5, MgSO_4_.7H_2_O 7.5, K_2_HPO_4_ 7.5, KH_2_PO_4_ 17.5, NaCl 2.5, EDTA-alkaline solution EDTA 50, KOH 31, Acidified Iron Solution FeSO_4_.7H_2_O 4.98, H_2_SO_4_ 1 mL, Boron Solution H_3_BO_3_ 11.42, Trace Metal Solution ZnSO_4_.7H_2_O 8.82, MnCl_2_.4H_2_O 1.44, MoO_3_ 0.71, CuSO_4_ 5H_2_O 1.57, Co(NO_3_)_2_ 6H_2_O 0.49 with a final pH of 6.6 ± 0.2. Incubation of Petri dishes was done at 25 °C ± 02, 16 h–8 h light–dark photoperiod cycle and irradiance of 100 µmol photons m^−1^ s^−1^ during 2 weeks. After the apparition of colonies, every single colony was purified on BBM agar plate until getting axenic culture. The bacterial contamination were prevented using streptomycine sulfate within the agar plate (Raja et al. 2007). The morphological features were observed using the optical microscope (S/N-EU 1900816, euromex, BioBlue Lab), and the scanning electron microscope (SEM) Quattro ESEM, Thermo Fisher Scientific–US, with a field emission gun.

### Growth conditions and exopolysaccharide extraction

The cultivation was carried out in BBM medium at 25 °C ± 02, under a white fluorescent light provided in 16 h–8 h light–dark photoperiod cycle, with an intensity of 150 µmol photons m^−1^ s^−1^. After cultivation, the culture were centrifuged at 4000 *g* for 30 min, the resulted free cell supernatant was concentrated by rotary evaporator (Heidolph Hei-VAP, Germany), at 45 °C for 100 ppm to about 1/4 of its initial volume, then, precipitated with cold ethanol (1:4 w/v) overnight at  − 20 °C. Another centrifugation was done at 6000 *g* for 10 min, the pellet was then collected, lyophilized (Alpha2-4 LSCbasic freeze dryer) and stored at 4 °C.

### Exopolysaccharide characterization

#### Biochemical analysis

The phenol–sulfuric acid technique was used to determine the total sugar content (TSC). TSC was calculated from the glucose calibration curve (Dubois et al. 1956). The protein content was determined using the Bradford assay with some modifications as reported in Berges et al. (1993); 1 mL (instead of 0.1 mL) of the standard, and 1 mL (instead of 3 mL) of Bradford reagent, then calculated from the BSA calibration curve. The modified Folin-Ciocalteu technique was also used to determine the total phenolic content (TPC). The gallic acid calibration curve was used to calculate the TPC (Bradford 1976; Berges et al. 1993; Zakaria et al. 2017; Kaliwal 2019).

#### Scanning electron microscopy coupled with energy dispersive X-ray analysis

The lyophilized powder of the crude extract was analyzed using a SEM (SEM, SIGMA 500/VP, ZEISS) at a voltage of 20 kV. The EPS is a non-conductor material, thus it was deposited onto carbon tape and gold-sputtered prior to analysis. The elemental composition on the EPS surface was determined using the Energy Dispersive X-Ray Analysis (EDX) technique (Fimbres-Olivarría et al. 2016; Olasehinde et al. 2019).

#### Fourier transform infra-red analysis

Fourier transform infrared (FT-IR) analysis was employed in the transmission mode at a range of 400–4000 cm^−1^, using a Perkin Elmer spectrometer at a resolution of 8 cm^−1^. The sample was compressed in a cold 150 Mpa isostatic press (CIP) with 23 ± 2 mg of KBr, to attain a 200–250 μm thick pellet (Ferreira et al. 2020).

### Gas chromatography mass spectrometry

The monosaccharide composition was determined using gas chromatography mass spectrometry (GC–MS) ‘Agilent 6890 Series GC System coupled to an Agilent 5973 Network, Shimadzu, GCMS-QP2020 NX’. GC–MS is a widely applied technique in the identification of polysaccharide composition due to high accuracy and repeatability (Zhang and Zhou 2017). Briefly, 10 mg of crude EPS were hydrolyzed using 1 mL of trifluoroacetic acid 2 M at 120 °C for 1 h 30 min. The liquid was then evaporated at 60 °C by nitrogen stream. Then, the hydrolysate was subjected to derivatization using (BSTFA/TMCS; 99%/1%), under Argon gas, the mixture was incubated at room temperature for 2 h to make trimethylsilyl-*O*-glycosides, which were then solubilized in dichloromethane after the reagent was evaporated. Next, the obtained derivation was injected to the separation column OPTIMA 1MS (30 m × 0.32 mm id, 0.25 μm) with a helium flow rate of 2.3 mL/min. Firstly, temperature was at 100 °C for 3 min. Secondly, an increment of 8 °C/min up to 200 °C for 1 min was used before a final increment of 5 °C/min up to 250 °C. The electronic impact (EI, 70 eV) ionization method was applied with the trap temperature set at 150 °C and the target ion was fixed at 40–800 m/z. Twelve mono-sugars were used as standards (Arabinose Ara, Rhamnose Rha, Galactose (Gal), Glucose (Glc), Galacturonic acid (GalA), Xylose (Xyl), Fucose (Fuc), Mannose (Man), Glucuronic acid (GlcA), Ribose (Rib), Glucosamine (GlcN) and Galactosamine (GalN) (Sigma-Aldrich).

### In vitro assessment of anti-α-d-glucosidase activity

The test measures the in vitro inhibition potential of the α-d-Glucosidase enzyme by the crude EPS, it is based on the measurement of the released ρ-nitrophenyl-α-d-glucopyranoside (ρ-NPG). Briefly, 0.5 mL of the α-d-glucosidase enzyme 0.2 U mL^−1^ (Sigma-Aldrich) was added to 0.1 mL of the EPS extract–previously suspended in H_2_O^–^ and was incubated at 37 °C for 15 min under gentle stirring. Next, 0.125 mL (4 mM) of ρ-NPG was added, then, incubated at 37 °C for 20 min. Afterward, 1 mL of Na_2_CO_3_ (0.2 M) was supplied to stop the reaction. Finally, the absorbance was read at λ = 405 nm using a spectrophotometer (6850 UV/Vis, JENWAY) (Bisht et al. 2013; Qian et al. 2015). The acarbose (Sigma-Aldrich) was used as a positive control. The percentage of inhibition was calculated as follows:

1 $$Inhibition\,\% = \frac{{\left( {ABS\,control - ABS\,sample} \right)}}{ABS\,control} \times 100$$

ABS: absorbance.

### Statistical analysis

Every measurement was made in triplicate, and results were expressed as means ± SD. The linear regression was used to determine the IC_50_ of the α-d-Glucosidase inhibitory effect of EPS extract using XLSTAT 2022 (trial version, Add in soft Inc., Boston, MA, USA).

## Results and discussions

### Physicochemical parameters measurement of Lake water

The measured parameters on Lake water of Chott Ain El-Beida are: potential of hydrogen (pH), temperature (°C), salinity (g L^−1^), electrical conductivity (mS^−1^ cm^−1^), dissolved oxygen (ppm) and total dissolved solids (ppt). These parameters give information on water characteristics and the conditions surrounding the microalgae flora in this ecosystem. The main observation concerns the high salinity (51.46 g L^−1^) and conductivity (74.70 mS^−1^ cm^−1^) of water (Table 1).

**Table 1:** Physicochemical parameters of Chott Ain El-Beida Lake

Parameters	Values
pH	6.64
Temperature (°C)	12.95
Salinity (g L^−1^)	51.46
Conductivity (m S^−1^ cm^−1^)	74.70
Dissolved oxygen (ppm)	0.54
Total dissolved solids (ppt)	36.98

Water physicochemical parameters provide some information on the microalgal native flora of the marine community, and aiding in the interpretation of different algal classes occurrence in their environment. The degree of salinity (Sn) makes it possible to classify water according to 4 categories: freshwater (Sn < 0.5 g L^−1^), freshwater to brackish (0.5 to 5 g L^−1^), brackish to salty water (18 to 30 g L^−1^) and salt water (Sn > 30 g L^−1^) (Hecker et al. 1996). Therefore, the water of Chott Aïn El-Beida Lake water is defined as hypersaline water. Saharan environments have been investigated as having little biological interest and limited biodiversity. However, this notion has changed recently, research has shown that arid habitats are home to unique species which adapt to the harshest ecosystems by modulating their physiology to acquire more resistance capacities (Guezoul et al. 2013).

### Microscopic observation and biochemical analysis

**Fig. 1:**
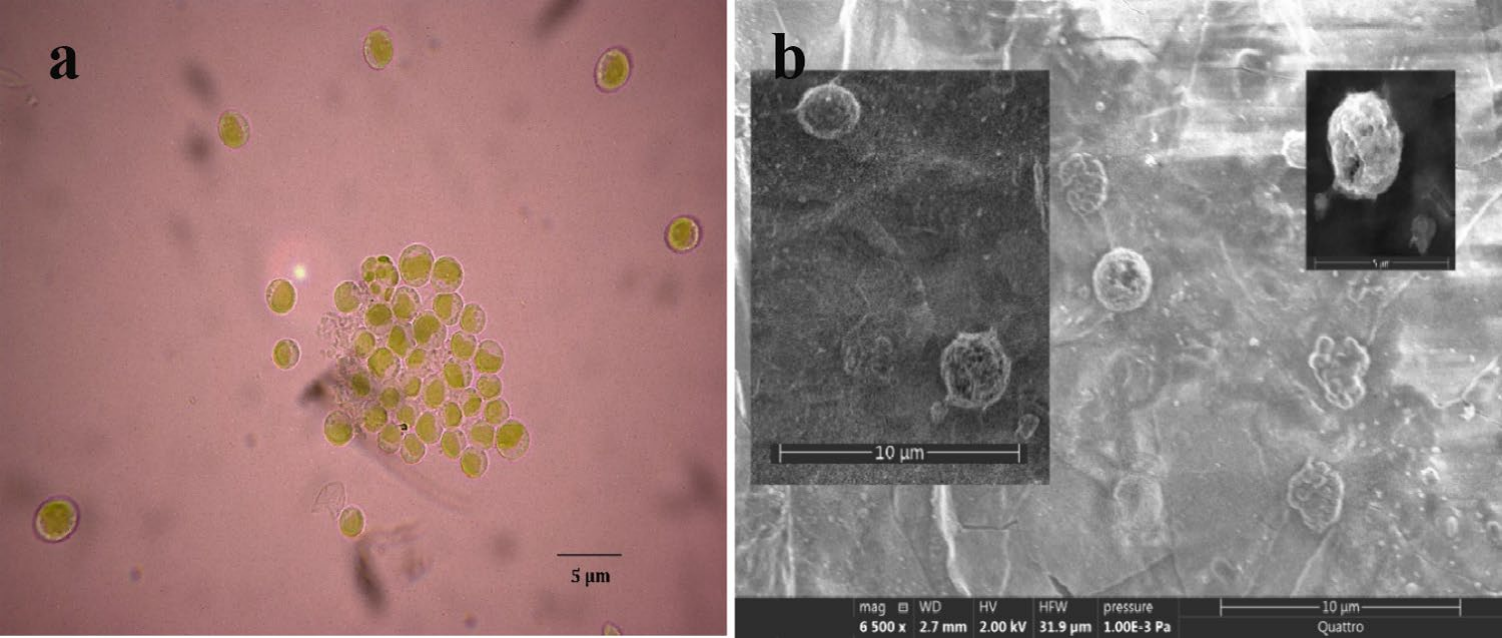
(**a**) *Chlorella* sp., (S1) cells observed under a light microscope × 100, (**b**) *Chlorella* sp. (S1) cells observed under a scanning electron microscope

A strain was isolated from Chott Aïn El-Beida Lake, situated in the Algerian desert. The microscopic observations and morphological features revealed the small green cells ranging between 2–10 µm in diameter that belong to *Chlorella* sp*.* (Fig. 1).

The biochemical analysis demonstrates different fractions of the extract (Table 2).

**Table 2:** Composition of the crude exopolysaccharides

Composition	Total sugar (% w/w)	Protein (%w/w)	Polyphenols (mg GAE g^−1^)
Crude EPS	65.26 ± 0.017	13.04 ± 0.011	0.267 ± 0.022

It is known that each fraction of the microalgae’s cell differs depending on the strain type and their physiological reactions to biotic and abiotic parameters (Barkia et al. 2019). In this study, the total sugar, the total protein, and the polyphenols amounts of the crude EPS were close to those obtained in other studies (Chen et al. 2016; Trabelsi et al. 2016; Kamble et al. 2018; Yu et al. 2019; Casas-arrojo et al. 2021), who described a total carbohydrate fraction of 48.53%, 57.53%, 52%, 52.74%, and 78%, and a total protein of 15.96%, 8.29%, 12%, 0.75%, and 2% from *Porphyridium cruentum, Chlorella vulgaris*, *Chlorella pyrenoidosa*, *Graesiella* sp., and *Chlamydomonas reinhardtii*, respectively.

### Exopolysaccharide characterization

**Fig. 2:**
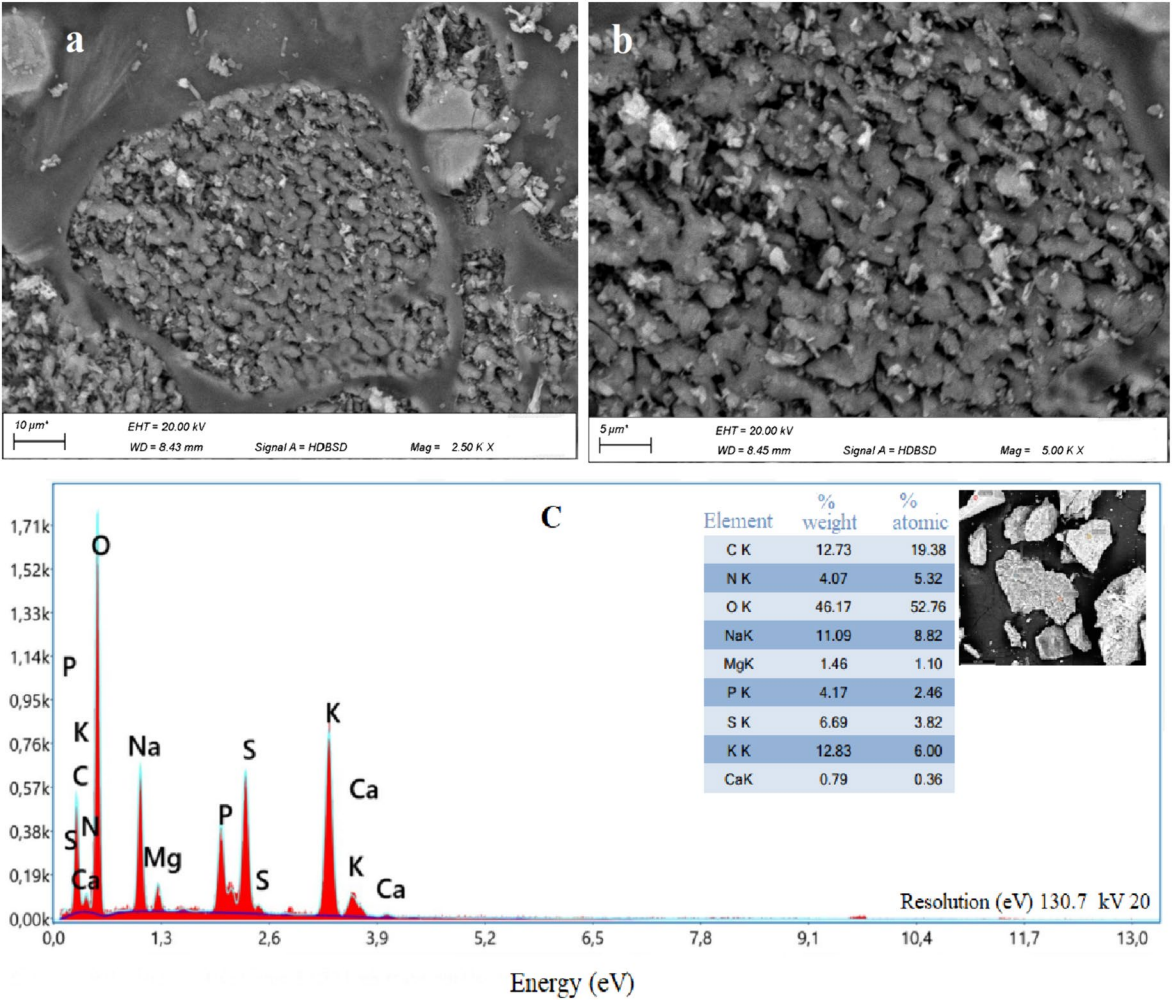
(**a** and **b**) Micrographs of the lyophilized EPS powder derived from *Chlorella* sp., obtained by SEM at different magnifications (A × 2.5k, B × 5k), (**c**) EDX showing different constitutive elements on the surface of the crude EPS

The extracted EPS consisted of a white-colored powder, with fine and granulated parts, the SEM micrographs showed an amorphous solid state, and granule forms, with compact and porous structure of irregular shape (Fig. 2a, b). The EDX analysis confirms the presence of sulfur (Fig. 2c). Sulfur is a common element in microalgal EPS, it is implicated in the functional groups, and contributes to the negative charges of these biomolecules. It was reported that the EPS produced by 120 marine microalgae were mostly sulfated (Raposo et al. 2014; Liu et al. 2016).

Sulfate quantity and its position in the EPS affects positively its bioactivity. Among the possible explanations is that the presence of a sulfate group in the EPS activates the hydrogen on the anomeric carbon, which increases the EPS’s hydrogen supply capacity and hence its potentiality. Also, a high sulfate content can increase the polysaccharide’s water solubility and improves its biological activity (Zhong et al. 2019). Sometimes an abnormally high sulfate content may affect the structure; the increasing in the degree of sulfation disrupts the EPS’s triple-helical structure, thus, reducing its capacity to supply hydrogen, resulting in less potentiality of the EPS (Liu et al. 2018).

In contrast to polysaccharides obtained from various organisms, microlgae-derived polysaccharides, referred to as sulfated polysaccharides due to the presence of sulfate esters, they exhibit diverse biological properties such as anticoagulant, anti-tumor, antidiabetic, anti-microbial, anti-radiation, anti-inflammatory effects… These sulfated polysaccharides have demonstrated efficacy in preventing the accumulation of free radicals and reactive chemical species, thereby serving as a protective mechanism against oxidative and radical stress agents (Arunkumar et al. 2021, 2023). The effectiveness of these polysaccharides is intricately tied to factors such as their degree and position of sulfation, sugar composition, molecular weight, and the treatment process (Delattre et al. 2016; Guehaz et al. 2024). Sulfate groups are incorporated into the sugar structure’s backbone to withstand harsh marine conditions such as high salinity, resulting in modifications to their polymeric structure, transforming into sulfated polysaccharides that exhibit significant biological activity and find extensive commercial applications (Muthukumar et al. 2021). The metabolism of the synthesis of the polysaccharide inside the microalgal cells, which enzymes are implicated, and how enzymes participate in the maturation of the final structure of the polysaccharides is yet to be clear. These mechanisms have a crucial relation to the phylogenic origin, however, herein further research is needed to elucidate the sulfation step in polysaccharides derived from microalgae, and how the degree and the position of sulfation is controlled.

**Fig. 3:**
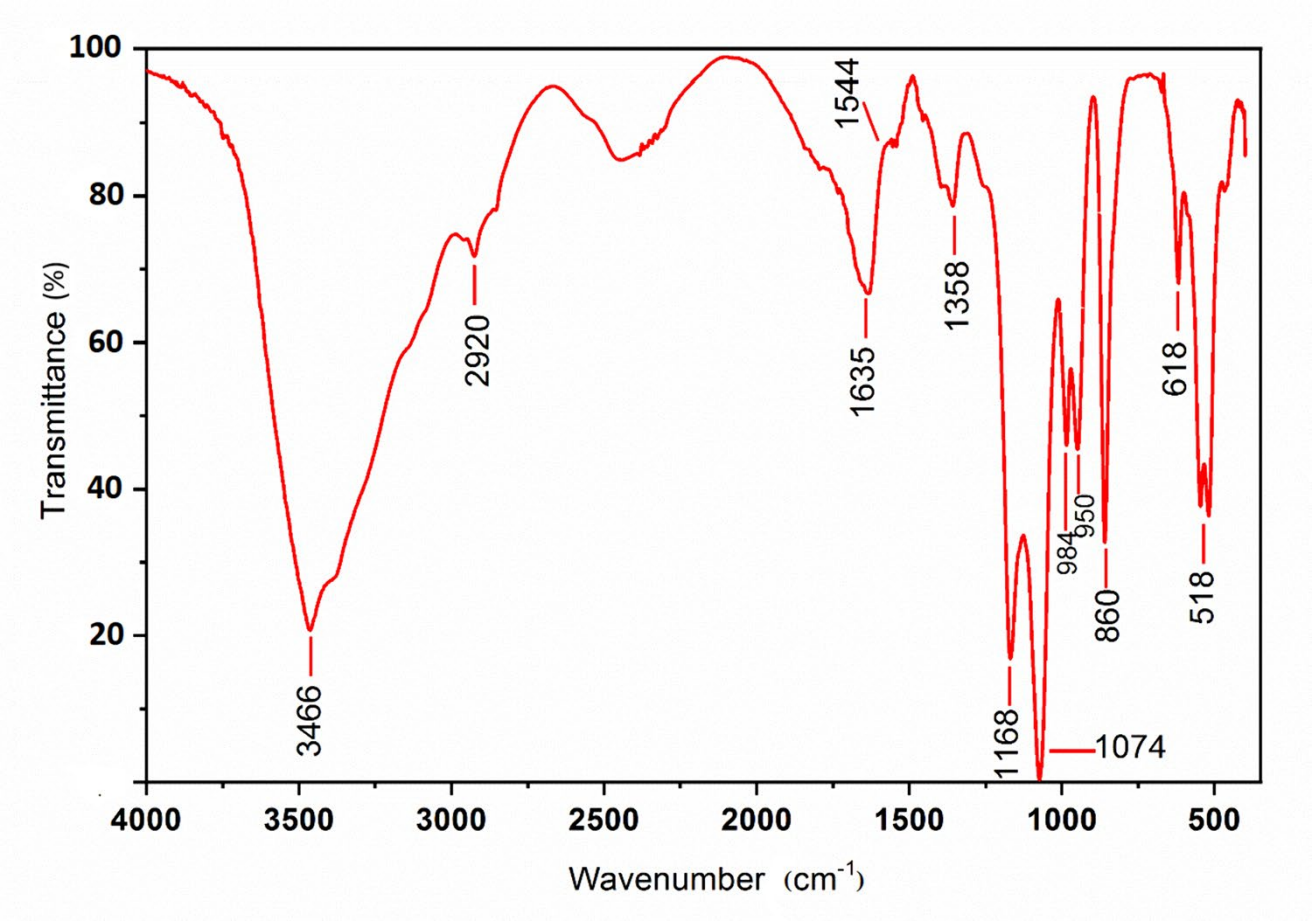
FT-IR spectrum of crude EPS extracted from *Chlorella* sp

The FT-IR revealed many peaks ranging from 3466 to 518 cm^−1^ (Fig. 3). The broad intense absorption peak around 3466 cm^−1^ and 2920 cm^−1^ characterizes the stretching vibration of O–H and C-H in the sugar residues, respectively (Sun et al. 2014; Song et al. 2018). The bands around 1635 cm^−1^ and 1358 cm^−1^ were possibly attributed to the carboxyl groups due to the presence of glucuronic acid (Xia et al. 2014). The absorption observed at 1168 cm^−1^ and 1074 cm^−1^ may be due to C–O–C stretching of glycosidic bands, and the presence of C-O–H side groups of the pyrane ring in the extracts, respectively (Wang et al. 2014; Chokshi et al. 2016). The weak band at 1263 cm^−1^ and the band at 860 cm^−1^ are assigned to the S = O and C-O-S stretching vibrations of the sulfate groups, respectively (Abd El Baky et al. 2014; Fimbres-Olivarría et al. 2016; Yuan et al. 2020). Bands at 984 cm^−1^ and 950 cm^−1^ were generally attributed to C–C and C-O stretching vibrations in pyranose rings and indicating the presence of polysaccharides as the major component (Malinowska et al. 2018).

Various methods were used for the characterization of the polysaccharides derived from microalgae and cyanobacteria, however, GC–MS remain the best method of choice (Delattre et al. 2016; Mehta and Shah 2021). The GC–MS technique revealed a heteropolysaccharide composed mainly of galactose 35.75%, glucose 21.13%, xylose 16.81%, rhamnose 11.57%, (Table 3). Jakhu et al. (2021) have reported similar results concerning the dominant sugars; galactose 33.43%, glucose 22.10%, and xylose11.86% when the product was derived from *Chlorella* sp. strain (Jakhu et al. 2021). According to Capek et al. 2020; galactose was the dominant hexose in the EPS isolated from *Chlorella vulgaris* with 37.9% (Capek et al. 2020). Also, galactose is the main constituent in the EPS of *Dictyosphaerium chlorelloides* with 42% (Halaj et al. 2022). Koçer et al. (2021) have studied the composition of the EPS of *Chlorella minutissima* and *Chlorella sorokiniana*; again, the main sugars were galactose 51.28%, 45.89% and glucose 25.07%, 28.68% respectively, the EPS from both strains contained a weak fucose content 0.12% and 1.33% respectively (Koçer et al. 2021). Kokarakis et al. (2022) reported different EPS composition from *Chlorella* sp.; glucosamine 23.4%, galactosamine 17.8%, rhamnose 25.5%, Arabinose 10.3%, glucose 8.9%, xylose 5.5% and mannose 2.7% (Kokarakis et al. 2022).

**Table 3:** Monosaccharide composition of the crude exopolysaccharide

Identified monosaccharides	Molar ratio (%)	Mass ratio (%)
Ara	5.10	4.47
Rha	11.57	11.11
Gal	35.75	37.67
Glc	21.13	22.26
Xyl	16.81	14.76
Fuc	6.96	6.68
GlcA	2.68	3.04

### In vitro assessment of anti-α-d-glucosidase activity

**Fig. 4:**
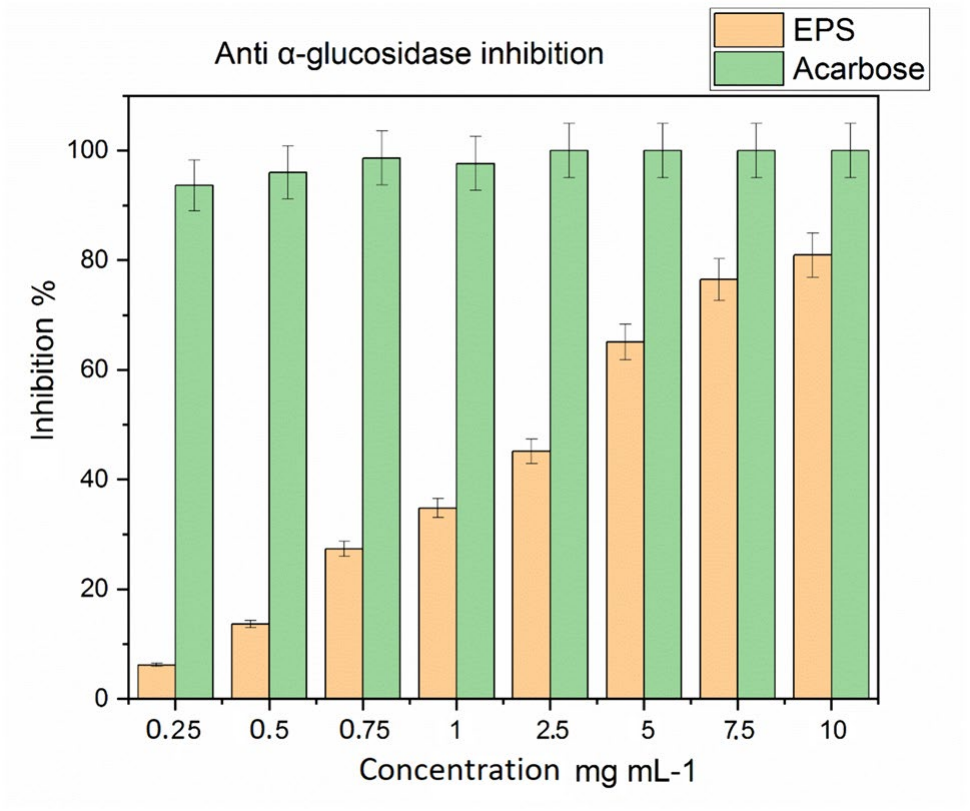
Inhibition of α-d-Glucosidase enzyme by crude EPS derived from *Chlorella* sp

The EPS extract was assessed for the in vitro inhibition of the α-d-Glucosidase enzyme, the results present the inhibition percentages of both acarbose and crude EPS at different dilutions (Fig. 4). The EPS inhibits α-d-Glucosidase enzyme in dose-dependent manner. The inhibition increases from 6.2% at 0.25 mg mL^−1^ to reach 80.94% at maximal concentration of 10 mg mL^−1^.

The half-maximal inhibitory concentration (IC_50_) was determined and made with that of acarbose (Table 4). The IC_50_ equals to 4.31 ± 0.20 mg mL^−1^ compared 0.39 ± 0.04 mg mL^−1^ of acarbose.

**Table 4:** IC_50_ values of *Chlorella* sp. EPS extract and acarbose against α-d-Glucosidase

Molecule	Acarbose	*Chlorella* sp. EPS
IC_50_ value (mg mL^**−**1^)	0.39 ± 0.04	4.31 ± 0.20

Our findings are close to those made on the methanolic extract of *Arthrospira platensis,* that reported a strong α-glucosidase inhibitory activity of 97.42% and an IC_50_ value of 9.56 mg mL^−1^ (Gheda et al. 2021). Likewise, ethyl acetate extracted from *Nannochloropsis oculata* exhibited a significant inhibitory action on the α-glucosidase enzyme by 80.42% at the concentration of 1 g mL^−1^ with an IC_50_ value of 178.53 μg mL^−1^ (Deepa et al. 2022).

Moreover, Priatni et al. (2016) have assessed the α-d-glucosidase inhibition performed by EPS from 10 different Indonesian cyanobacterial producers. Although most of the EPSs exhibited inhibitory activity, there values were lower compared to the results from the current study. The highest inhibitory levels were exhibited by EPSs from *Pseudanabaena* sp. (14.02%) and *Chroococcus* sp. (13.0%) isolates, while products from *Phormidium* sp. and *Oscillatoria limnetica* did not exhibit any inhibitory activity. Additionally, Qi and Kim (2017) have investigated the α-d-glucosidase activity of carotenoids derived from *Chlorella ellipsoidea*. The extracts displayed potent inhibitory effect against α-d-glucosidase with a non-competitive inhibition (Qi and Kim 2017). In addition, fucoxanthin from *Phaeodactylum tricornutum* exerted a weak inhibitory activity against rat-intestinal α-glucosidase 32.18% at a concentration of 1 g mL^−1^ in a dose-dependent manner with an IC_50_ value of 4.75 µg mL^−1^ (Arthitaya et al. 2019).

## Conclusion

While the utilization of microalgae or their byproducts as food alternatives is not yet competitive, this research underscores the importance of exploring harsh environments that may harbor native strains producing bioactive compounds crucial for developing nutritious and health-promoting foods, especially in the context of diabetes.

This preliminary investigation presents findings on the extraction of an EPS derived from *Chlorella* sp. strain found in the hypersaline Chott Aïn El-Beida Lake within the Algerian Sahara. The study highlights the notable potential of this EPS as an anti-hyperglycemic agent. The composition of the EPS includes galactose, glucose, xylose, rhamnose, fucose, arabinose, and glucuronic acid, accompanied by sulfate, methyl, and carboxyl functional groups. The observed efficacy is attributed to the sulfate and glucuronic acid content.

Although this study contributes valuable insights into the anti-hyperglycemic activity of *Chlorella* sp.-derived EPS, further research is imperative to fully characterize the EPS’s structure and optimize growth conditions. Additionally, assessing the EPS’s potential through in vivo investigations using animal models, exploring additional parameters, and elucidating the mechanistic processes involved in diabetes modulation will be worthwhile.

## Data Availability

The datasets generated and/or analyzed during the current study are available from the corresponding author on reasonable request.

## Footnotes

1 (European Union, Novel Food catalogue & No 2015/2283, EFSA).
